# Artificial intelligence based surgical support for experimental laparoscopic Nissen fundoplication

**DOI:** 10.3389/fped.2025.1584628

**Published:** 2025-05-23

**Authors:** Holger Till, Ciro Esposito, Chung Kwong Yeung, Dariusz Patkowski, Sameh Shehata, Steve Rothenberg, Georg Singer, Tristan Till

**Affiliations:** ^1^ Department of Pediatric and Adolescent Surgery, Medical University of Graz, Graz, Austria; ^2^ Division of Pediatric Surgery, Federico II University Hospital, Naples, Italy; ^3^ Department of Surgery, University of Hong Kong, Hong Kong, China; ^4^ Department of Pediatric Surgery and Urology, Wroclaw Medical University, Wroclaw, Poland; ^5^ Department of Pediatric Surgery, University of Alexandria, Alexandria, Egypt; ^6^ Department of Pediatric Surgery, Rocky Mountain Hospital for Children, Denver, CO, United States; ^7^ College of Computing and Data Science (CCDS), Nanyang Technological University, Singapore, Singapore

**Keywords:** artificial intelligence (AI), computer vision (CV), visual quality indicators (VQIs), Nissen fundoplication, EfficientNet

## Abstract

**Background:**

Computer vision (CV), a subset of artificial intelligence (AI), enables deep learning models to detect specific events within digital images or videos. Especially in medical imaging, AI/CV holds significant promise analyzing data from x-rays, CT scans, and MRIs. However, the application of AI/CV to support surgery has progressed more slowly. This study presents the development of the first image-based AI/CV model classifying quality indicators of laparoscopic Nissen fundoplication (LNF).

**Materials and methods:**

Six visible quality indicators (VQIs) for Nissen fundoplication were predefined as parameters to build datasets including correct (360° fundoplication) and incorrect configurations (incomplete, twisted wraps, too long (>four knots), too loose, too long, malpositioning (at/below the gastroesophageal junction). In a porcine model, multiple iterations of each VQI were performed. A total of 57 video sequences were processed, extracting 3,138 images at 0.5-second intervals. These images were annotated corresponding to their respective VQIs. The EfficientNet architecture, a typical deep learning model, was employed to train an ensemble of image classifiers, as well as a multi-class classifier, to distinguish between correct and incorrect Nissen wraps.

**Results:**

The AI/CV models demonstrated strong performance in predicting image-based VQIs for Nissen fundoplication. The individual image classifiers achieved an average F1-Score of 0.9738 ± 0.1699 when adjusted for the optimal Equal Error Rate (EER) as the decision boundary. A similar performance was observed using the multi-class classifier. The results remained robust despite extensive image augmentation. For 3/5 classifiers the results remained identical; detection of incomplete and too loose LNFs showed a slight decline in predictive power.

**Conclusion:**

This experimental study demonstrates that an AI/CV algorithm can effectively detect VQIs in digital images of Nissen fundoplications. This proof of concept does not aim to test clinical Nissen fundoplication, but provides experimental evidence that AI/CV models can be trained to classify various laparoscopic images of surgical configurations. In the future, this concept could be developed into AI based real-time surgical support to enhance surgical outcome and patient safety.

## 1 Introduction

Computer vision (CV), a branch of artificial intelligence (AI), enables deep learning models to detect and interpret specific events in digital images or videos through prior training. Especially in medical imaging, AI/CV holds significant promise analyzing data from x-rays, CT scans, and MRIs. In surgery, AI/CV could support intraoperative decision-making, reduce errors, and potentially improve patient outcomes by providing real-time feedback on procedural quality indicators (1).

Despite growing interest in AI and CV applications in medicine, the development of effective models specifically tailored to videoscopic surgery remains limited. To date, only a few AI and CV models have been designed to support surgical quality assurance, with the primary aim on recognizing procedural steps and performance indicators in real-time mainly focusing on laparoscopic cholecystectomies (2, 3).

One reason for this slow development could be that while highly skilled surgeons are best equipped to define which visible quality indicators (VQIs) are most relevant to patient outcomes, many lack the AI/CV expertise necessary to engineer the development of such algorithms (4). Conversely, AI engineers may not fully understand the nuances of surgical procedures, making an intimate collaboration between these two fields essential.

The Nissen fundoplication represents a well-established surgical procedure to treat gastroesophageal reflux disease (GERD), particularly when medical management with acid suppressants and lifestyle changes fail to provide sufficient relief (5, 6). A “correct” Nissen procedure involves a 360° wrap of the gastric fundus around the lower esophagus, creating a one-way valve that enhances the function of the lower esophageal sphincter (7). Vice versa, a Nissen wrap would be considered “incorrect” when the wrap is incomplete, twisted, too long, too loose, or positioned below the gastroesophageal junction. Such visible features of the wrap could serve as quality indicators. However, the question arises whether AI/CV algorithms could be trained with images of VQIs to detect such events in a different testset. To date, no AI algorithm has been developed specifically for surgical support of laparoscopic Nissen fundoplication and it is not known whether AI models can differentiate between correct and incorrect Nissen wraps. In this study, we therefore aimed to examine whether an AI model can effectively detect VQIs in digital images of Nissen fundoplications in an experimental model and present the first AI/CV algorithm specifically designed for laparoscopic Nissen fundoplication (LNF), built upon a comprehensive set of VQIs.

## 2 Material and methods

### 2.1 Concept

Six visible quality indicators (VQIs) for Nissen fundoplication were predefined as parameters to build datasets and train the AI/CV algorithm including correct (360° fundoplication) and incorrect configurations (incomplete, twisted wraps, too long (more than four knots), too loose or malpositioning (at/below the gastroesophageal junction).

### 2.2 Data collection

Following approval by the responsible veterinary board (2020-0-814-140) for a porcine experiment (one animal) three pediatric surgical residents and three attending surgeons each performed 10 iterations of the procedure. After each iteration, the sutures were reopened and the fundus was repositioned to its original position. Each procedure was recorded in short video segments from various angles and distances, with footage captured at 30 frames per second to ensure comprehensive visual data.

### 2.3 Model development

**Figure 1:**
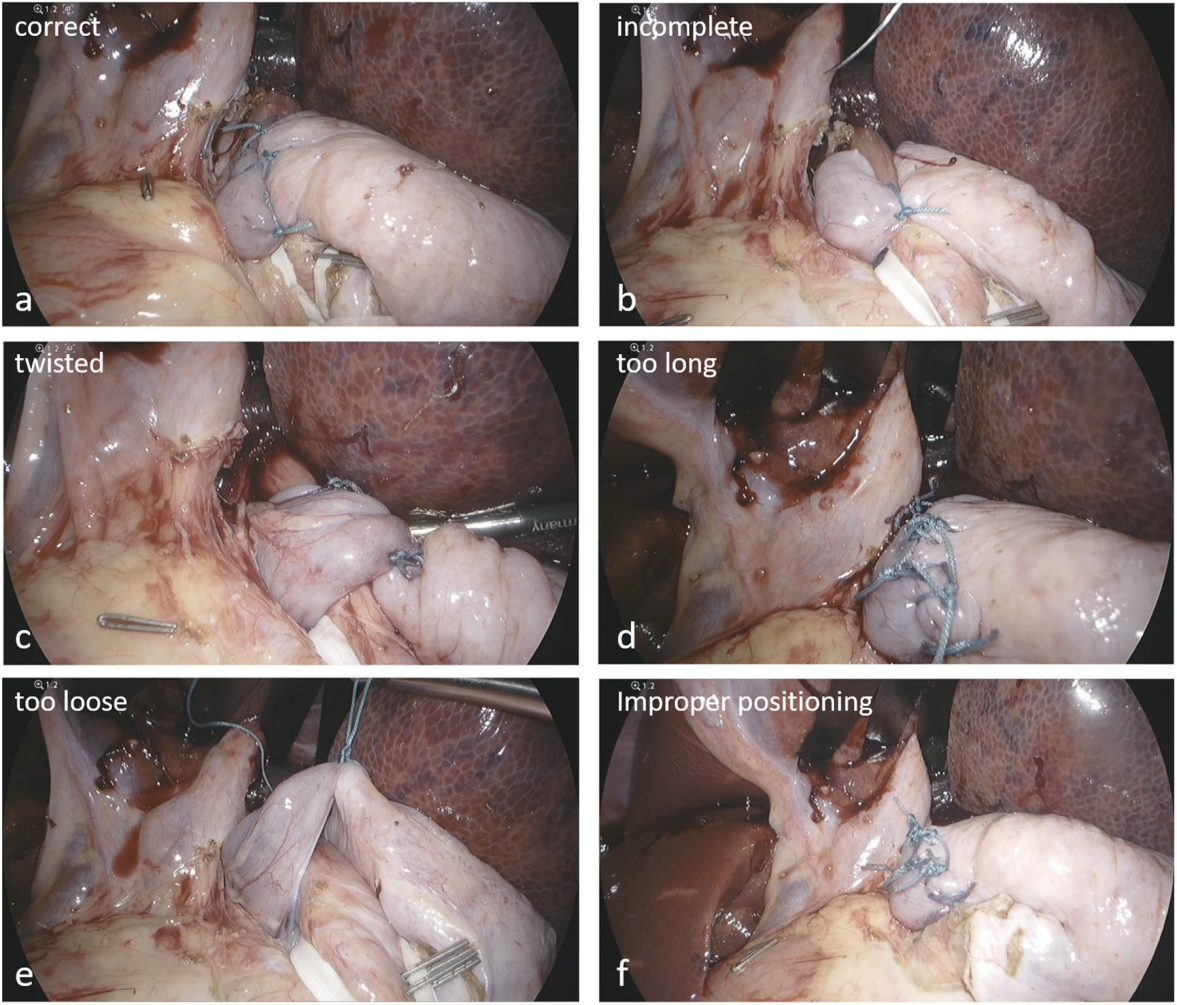
Representative examples of annotated VQIs. **(a)** correct configuration, **(b)** incomplete, **(c)** twisted, **(d)** too long, **(e)** too loose, **(f)** malpositioning below the GE junction.

Three video sequences had to be excluded for technical reasons. Therefore, a total of 57 video sequences were processed. Initially, frame-based volume filtering was conducted by medical experts, selecting none, one or multiple images from an array of individual frames in every 0.5 s interval within the videos. The selection process aimed at discarding irrelevant (no Nissen visible), inconclusive (Nissen not complete), non-representative (Nissen visible, but does not show key characteristics of VQIs or complete Nissen), or redundant (very similar or identical images in a selection) data points. Afterwards, images were annotated on their respective VQIs using class labels. Due to the filtering procedure, all images not labelled with one of the VQIs were deemed to be correct. Completing this procedure yielded a total of 3,138 fully annotated images (Figure 1). All images were then divided randomly into training (70%), validation (20%), and testing (10%) sets (Tables 1, 2). For certain experiments, data was augmented to avoid overfitting and increase representation learning, adding random vertical flips, rotations, color jitter and resizing to images during training and validation.

**Table 1:** Total number and relative percentage of data samples within the training, validation and test sets.

		Training		Validation		Testing		Total
Total		2,431	69.46%	705	20.14%	364	10.40%	3,500
Label	Incomplete	311	66.17%	101	21.49%	58	12.34%	470
	Twisted	274	71.54%	76	19.84%	33	8.62%	383
	Too long	345	66.99%	102	19.81%	68	13.20%	515
	Too loose	40	60.61%	17	25.76%	9	13.63%	66
	Below GE junction	261	65.74%	83	20.91%	53	13.35%	397
	Correct	1,200	71.90%	326	19.53%	143	8.57%	1,669

Images with more than 1 VQI are counted separately; hence, the total exceeds the 3,138 samples.

**Table 2:** Total number and relative percentage of samples per class during training for the ensemble of classifiers, as well as the multi-class classifier.

		Training		Validation		Testing	
Labels	Incomplete	311	20.58%	101	23.65%	58	28.86%
	Correct	1,200	79.42%	326	76.35%	143	71.14%
	Twisted	274	18.59%	76	18.91%	33	18.75%
	Correct	1,200	81.41%	326	81.09%	143	81.25%
	Too long	345	22.33%	102	23.83%	68	32.23%
	Correct	1,200	77.67%	326	76.17%	143	67.77%
	Too loose	40	3.23%	17	4.96%	9	5.92%
	Correct	1,200	96.77%	326	95.04%	143	94.08%
	Below GE junction	261	17.86%	83	20.29%	53	27.04%
	Correct	1,200	82.14%	326	79.71%	143	72.96%
	Incomplete	311	12.79%	101	14.33%	58	15.93%
	Twisted	274	11.27%	76	10.78%	33	9.07%
	Too long	345	14.19%	102	14.47%	68	18.68%
	Too loose	261	10.74%	83	11.77%	53	14.56%
	Below GE junction	40	1.65%	17	2.41%	9	2.47%
	Correct	1,200	49.36%	326	46.24%	143	39.29%

Class distribution may vary between training, validation and test sets due to random sampling.

The EfficientNet architecture (8) was utilized for image classification, optimizing the model to accurately distinguish between correct and incorrect Nissen fundoplication based on the annotated VQIs (9, 10). EfficientNet was selected as it represents a well-established and well-documented baseline across various benchmarks and tasks, providing a solid foundation upon which to build and evaluate this proof-of-concept (8). Models were trained for 50 epochs (10) with an initial learning rate of 0.01 and an Adam optimizer (11). No scheduler, weight-decay or momentum was configured beyond the default values. All classifiers used variations of cross-entropy loss for loss calculation. The multi-class classifier omitted the softmax operation within the activation layer in favor of a sigmoid activation function. For reasons of numerical stability, the pytorch BCELossWithLogits was used to simulate this behavior.

## 3 Results

**Figure 2:**
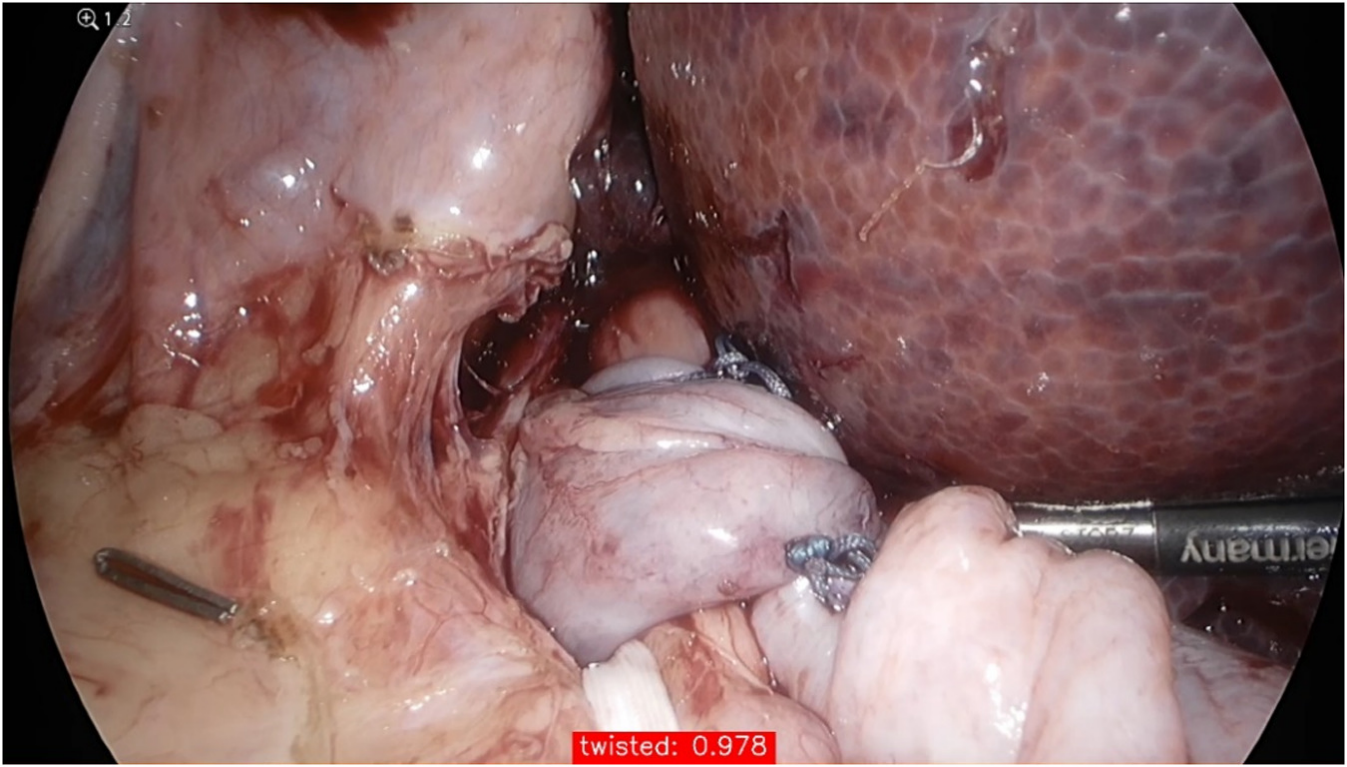
The confidence of the model for predicting a twisted wrap during testing; its performance is given in the red box at the bottom of the image (97.8%).

The AI/CV models exhibited strong performance in predicting image based VQIs for Nissen fundoplication. Individual image classifiers reported an average F1-Score of 0.9738 ± 0.1699 when adjusted for optimal Equal Error Rate (EER) as decision boundary, i.e., the model confidence, at which a true or false prediction is made (Figure 2). Similar results were achieved using a single multi-class classifier.

**Figure 3:**
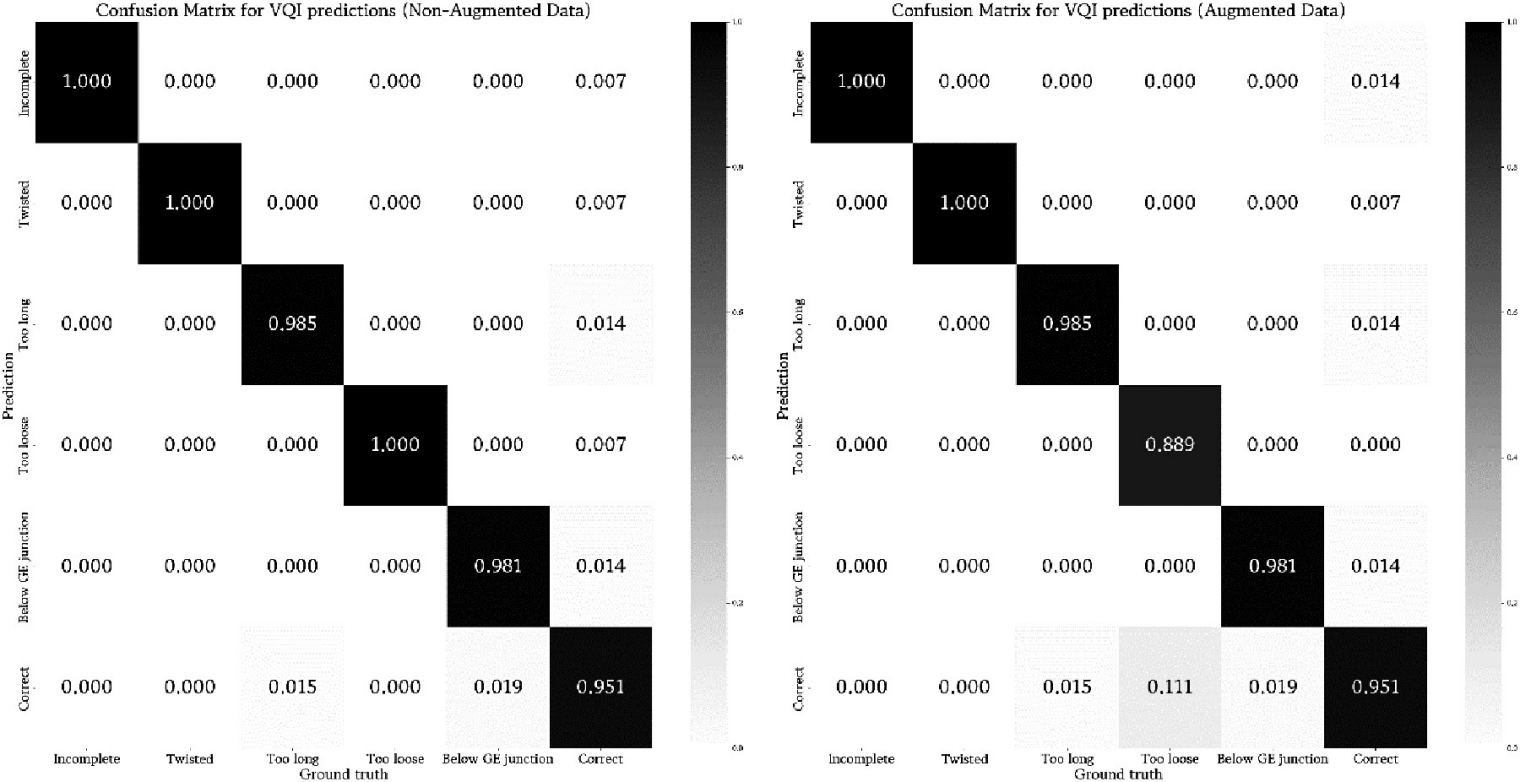
Visualization of the rate of true and false predictions for the ensemble of classifiers across all test samples (non-augmented and augmented data) displaying the average performance when tested on their respective VQI or correct samples. The number of samples was normalized between zero and one to enable inter-classifier comparability.

These results barely changed when applying considerable image augmentation techniques, as described in the methods section (Figure 3). For 3/5 classifiers the results remained identical, merely detection of incomplete and too loose Nissen wraps showed a slight decline in predictive power. Table 3 summarizes the performance metrics evaluated for the conducted experiments.

**Table 3:** Summary of crucial performance metrices of the ensemble of classifiers.

			Precision	Recall	Accuracy	F1-score
Average		Default	.9932 ± .009	.9571 ± .032	.9906 ± .005	.9738 ± .017
		Augmented	.9711 ± .047	.9739 ± .015	.9896 ± .004	.9719 ± .018
Model (vs. correct)	Incomplete	Default	1.000	.9810	.9950	.9915
		Augmented	1.000	.9650	.9900	.9831
	Twisted	Default	1.000	.9706	.9943	.9851
		Augmented	1.000	.9706	.9943	.9851
	Too long	Default	.9853	.9710	.9858	.9781
		Augmented	.9853	.9710	.9858	.9781
	Too loose	Default	1.000	.9000	.9934	.9474
		Augmented	.8890	1.000	.9934	.9412
	Below GE junction	Default	.9811	.9630	.9847	.9720
		Augmented	.9811	.9630	.9847	.9720

Every model was tested on either a sample containing a failed VQI or a correct image. Details can be found in Table 2.

**Figure 4:**
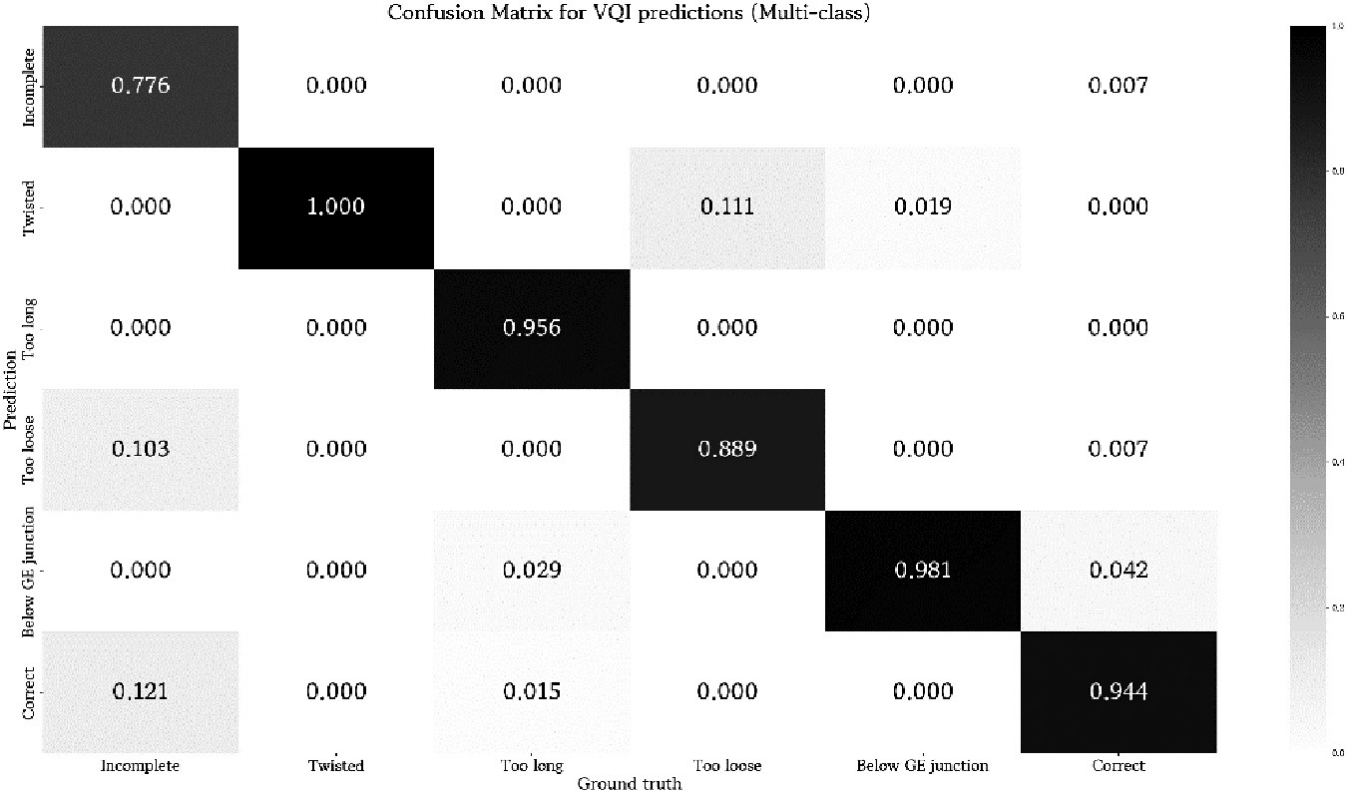
Confusion matrix for prediction results of the multi-class classifier. Values are normalized between zero and one with respect to the ground truth label of a test sample.

The results for multi-class classification, as displayed in Table 4; Figure 4, were only partly comparable to the ensemble of classifiers, as the former greatly limited the possibility of false negatives by turning every decision into a binary prediction. Nonetheless, the multi-class classifier showed satisfactory performance on the respective test set regardless. Only the results for augmented data were evaluated. Merely the true positive rate for incomplete Nissen decreased notably.

**Table 4:** Results for the measures precision, recall, accuracy and F1-score for multi-class classification.

			Precision	Recall	Accuracy^a^	F1-score
Average		Aug	.9365 ± .061	.9243 ± .075	.9284 ± .000	.9267 ± .035
Multi-class model	Incomplete	Aug	.9783	.7759	–	.8677
	Too long	Aug	.8904	.9559	–	.9220
	Too loose	Aug	1.000	.8889	–	.9412
	Twisted	Aug	.8250	1.000	–	.9041
	Below GE junction	Aug	.9811	.9808	–	.9809
	Correct	Aug	.9441	.9441	–	.9441

^a^ As True Negatives (TN) cannot be established on a per-class basis, accuracy is only given for the entire model.

## 4 Discussion

The integration of artificial intelligence (AI) and computer vision (CV) into surgery presents a complex and evolving endeavor, largely due to the variability in surgical environments, anatomical differences, and visual representation during individual procedures. For AI and CV to make a significant impact on surgery, it is essential to define procedure-specific visible quality indicators that the algorithm can be trained with in order to predict such features in other images.

CV and deep learning, a subset of AI, hold tremendous potential in laparoscopic surgery, which heavily relies on digital imaging. Various authors have already published AI applications to support recognition of instruments (12), phase (13), anatomy (12) and action (14). Nevertheless, the number of underlying procedures is still limited focusing on cholecystectomy (2, 3, 15–19) and gynecological procedures (hysterectomy and myomectomy) (12, 14).

A recently published report analyzing a total of 47 frames from 25 laparoscopic cholecystectomy (LC) videos has demonstrated that AI can be used to identify safe and dangerous zones of dissection within the surgical field. The authors could describe a high specificity/positive predictive values for Go zones and high sensitivity/negative predictive values for No-Go zones (15). The underlying algorithm, termed *GoNoGoNet* by the authors, is based on 2,627 random frames from 290 LC videos; the results of this study suggest that deep learning can be used to identify safe and dangerous zones of dissection and other anatomical structures such as the liver, gallbladder and hepatocystic triangle in the surgical field during LC with a high degree of performance (3). Similarly, Tokuyasu and coworkers have created an AI algorithm for object detection during LC to mitigate the risk of bile duct injury (16). They annotated approximately 2,000 endoscopic images of Calot's triangle and trained the YOLOv3 (You Only Look Once, version 3) algorithm, which successfully identified four critical landmarks during LC, thereby providing intraoperative indications that improved procedural safety (16). Kawamura et al. also have developed an AI system aimed at enhancing surgical safety during LC (2). Utilizing 72 LC videos, they annotated 23,793 images and trained their AI model based on performance metrics such as precision, recall, F-measure and specificity. Their model achieved an impressive overall precision of 0.971, demonstrating that AI/CV systems could effectively enhance surgical safety by delivering real-time visual feedback during operations.

In the context of laparoscopic distal gastrectomy, several articles report the implementation of AI models to recognize different surgical phases and workflows, further improving decision-making support during procedures (13, 20).

The above-mentioned examples indicate that AI models tailored to specific videoscopic procedures could significantly enhance intraoperative guidance, surgical outcome and safety. However, models focused on more complex procedures, such as Nissen fundoplication, still remain underdeveloped. Creating these models requires extensive international data collection and close collaboration between AI experts and videoscopic surgeons in order to collect a sufficient number of videos as well as to define appropriate VQIs. One of the associated problems may also be that the collection of datasets comprising incorrect images and videos of LNFs will most likely be difficult.

In our experimental study, we introduce the first AI/CV model designed to detect specific visible quality indicators in images of LNF. The selection of such parameters to build the algorithm was focused less on clinical importance or procedural complications, but on the visibility of such quality features in surgical images. We aimed to test whether the AI/CV algorithm can be trained with different shapes and features of Nissen wraps and whether the AI/CV can predict the presence of such VQIs in a different set of images (testset). While the scientific evidence of the chosen parameters is relatively scarce, the importance of these parameters for a successful laparoscopic Nissen fundoplication has been described in several reports (21–24). For instance, the importance of an appropriate orientation and positioning of the wrap above the GE junction has been described by Rothenberg in his report describing a 20-year experience with nearly 2,000 consecutive laparoscopic Nissen fundoplications (21). Moreover, a wrap that is too loose is incompetent to prevent reflux (25). If the wrap is too long then the passage of food can be obstructed (22). Nevertheless, our study does not claim clinical relevance yet, as it merely proves the experimental concept that image based events such as VQIs for LNF can be detected by an AI algorithm.

While our proof of concept is promising, it is important to clearly acknowledge the limitations of our experimental study. One key concern is the transferability of AI/CV models trained on animal data to human applications, which does not seem valid primarily. Indeed, some recent studies have highlighted the challenges of generalizing models across species due to anatomical and physiological differences. On the other hand, Wang et al. employed a U-Net model for neuroimaging trained on humans and later adapted for non-human primates (26). Their approach demonstrated that transfer-learning processes can enable models trained on one species to be effectively updated for use with scans from different species, such as macaques, chimpanzees, marmosets, and pigs (26). Further research, however, seems necessary in surgery to determine how animal-based training datasets can be adapted or augmented to ensure reliable application in humans.

The reason for choosing an animal model seems worthwhile emphazing. In order to build an AI/CV algorithm supporting surgical decision making it seems important to train the AI/CV with images depicting “right and wrong”. However, adequately sized datasets of “incorrect” Nissen wraps in humans are unlikely to be attained. Thus, the development of AI/CV models in humans seems unlikely in the near future. Instead, our experimental design provided access to numerous videos of incomplete, twisted, excessively long (more than four knots), too loose wraps, and positioning at or below the GE junction.

Another limitation remains the specific selection of the predefined VQIs. The rational for defining such visible quality parameters have been mentioned before. The purpose of this study was to see whether an AI algorithm can differentiate between different configurations of the Nissen wrap. The purpose of the study is not to cover all steps of a perfect antireflux surgery (e.g., dissection, extent of esophagophrenical mobilization and cruroplasty). Finally, we refrained from using tightness as it is rather a tactile and not a visual quality.

Thus, our AI/CV model exhibited strong performance in predicting image based VQIs for Nissen fundoplication and further research must address key issues such as robustness, potential biases, model explainability to fully eliminate any concerns regarding overfitting or memorization during training.

We have chosen EfficientNet as the primary model for this study due to its exceptional performance in image classification tasks while maintaining computational efficiency. EfficientNet has consistently demonstrated state-of-the-art performance across a range of image classification benchmarks (27, 28). Its ability to achieve high accuracy with relatively fewer parameters makes it an ideal choice for our task of detecting correct vs. incorrect configurations of Nissen wraps, where precision is critical. Given the nature of our dataset (focused on animal-based configurations) and the computational constraints, EfficientNet offers an excellent balance between model size, training time, and inference speed. This efficiency is crucial for practical applications, especially in environments with limited computational resources. Since the current work represents an experimental proof-of-concept testing whether an AI algorithm can classify various surgical reconstructions (configurations of Nissen wraps based on laparoscopic images), we did not optimize the algorithm yet by comparing EfficientNet performance with other models. Future work may now be fostered to evaluate the complexity of using alternative models like Vision Transformers (ViT) or Swin Transformers—which may require more data and computational power (29, 30). Moreover, explainability methods are key to induce trust and should be considered once transitioning from a proof-of-concept to a model used in surgical practice. Afterwards, the model architectures can be optimized to not only maximize predictive power but do so in a manageable scope for real-time classification. For AI models in general, also rigorous external validation, careful consideration of ethical issues, data security and privacy as well as education and training programs for surgeons and healthcare professionals are of upmost importance (31).

In conclusion, our proof of concept study represents a significant experimental advancement toward leveraging AI and CV technologies to enhance procedural quality assurance and optimize surgical outcomes. For the first time, an AI/CV model has been trained to recognize image based VQIs for Nissen fundoplication. Certainly, this study does not claim any clinical relevance yet, but may inspire further research of image based AI algorithms supporting surgical decision making.

## Data Availability

The raw data supporting the conclusions of this article will be made available by the authors, without undue reservation.
